# Spatial Isolation and Temporal Variation in Fitness and Condition Facilitate Divergence in a Migratory Divide

**DOI:** 10.1371/journal.pone.0144264

**Published:** 2015-12-14

**Authors:** Claudia Hermes, Raeann Mettler, Diego Santiago-Alarcon, Gernot Segelbacher, H. Martin Schaefer

**Affiliations:** 1 Department of Evolutionary Biology and Animal Ecology, Faculty of Biology, University of Freiburg, Germany; 2 Biología y Conservación de Vertebrados, Instituto de Ecología A.C., Xalapa, Mexico; 3 Department of Wildlife Ecology and Management, Faculty of Environment and Natural Resources, University of Freiburg, Germany; University of Arkansas, UNITED STATES

## Abstract

A novel migratory polymorphism evolved within the last 60 years in blackcaps (*Sylvia atricapilla*) breeding sympatrically in southwestern Germany. While most individuals winter in the traditional areas in the Mediterranean, a growing number of blackcaps started migrating to Britain instead. The rapid microevolution of this new strategy has been attributed to assortative mating and better physical condition of birds wintering in Britain. However, the isolating barriers as well as the physical condition of birds are not well known. In our study, we examined whether spatial isolation occurred among individuals with distinct migratory behaviour and birds with different arrival dates also differed in physical and genetic condition. We caught blackcaps in six consecutive years upon arrival on the breeding grounds and assigned them via stable isotope analysis to their wintering areas. Analysis of the vegetation structure within blackcap territories revealed different microhabitat preferences of birds migrating to distinct wintering areas. Blackcaps arriving early on the breeding grounds had higher survival rates, better body condition and higher multilocus heterozygosities than later arriving birds. We did however not find an effect of parasite infection status on arrival time. Our results suggest that early arriving birds have disproportionate effects on population dynamics. Allochrony and habitat isolation may thus act together to facilitate ongoing divergence in hybrid zones, and migratory divides in particular.

## Introduction

Sympatric speciation, the evolution of a barrier to gene flow within a panmictic and spatially unsegregated population, has been one of the most controversially discussed topics in evolutionary biology [1]. For long, it has been considered to be unlikely that reproductive isolation can arise despite the homogenizing effect of gene flow [1]. However, disruptive selection can lead to stable polymorphisms and promote the mechanism of assortative mating, where mating partners have more similar phenotypes than expected by random mating [2]. Under this assumption, a population can diverge into two ecologically isolated sub-populations [3] which might lead to speciation in sympatry [2].

Ecological isolation is facilitated by two possible premating barriers preventing gamete transfer between diverging populations: spatial and temporal isolation. Habitat segregation occurs when distinct sub-populations occupy different microhabitats within the same general area [4]. If the association between habitat preference and mating preference is sufficiently strong, random mating can give way to habitat-based assortative mating, and a formerly homogenous population is split into two isolated sub-populations specialized on distinct habitats [5]. Simulation studies suggest that sympatric speciation is a likely outcome of disruptive selection on habitat preference [5]. This has been shown in the apple maggot (*Rhagoletis pomonella*), where populations diverged ecologically after a host shift [6]. In birds, however, sympatric speciation is generally rare due to their often high dispersal rates, which facilitate gene flow across habitat boundaries. It is estimated that only about 5% of all bird species have evolved sympatrically [7].

Nevertheless, habitat isolation might influence the dynamics of the hybrid zone of two ecologically very similar species, the pied flycatcher (*Ficedula hypoleuca*) and the collared flycatcher (*F*. *albicollis*) [8]. If fledglings imprint on their natal microhabitats, they may return to breed in the same habitat. Such imprinting would promote spatial isolation. Importantly, imprinting can promote divergence and speciation by intensifying the strength of disruptive selection [9]. Similarly, spatial segregation by different microhabitat choice is implicated as the key mechanism for divergence in eight sympatric leaf warbler species in the genus *Phylloscopus* [10]. Allochrony, that is temporal isolation, can lead to competition for the optimal microhabitat, forcing later breeding individuals to make a suboptimal choice and therefore driving habitat segregation, as it was suggested for the common cuckoo *Cuculus canorus* [11].

Allochrony has been shown to promote assortative mating and reproductive isolation in the Madeiran storm petrel (*Oceanodroma castro*) on the Azores [12] and also occurs in the hybrid zone of Swainson’s thrush (*Catharus ustulatus*) [13]. Avian migration can lead to temporal isolation, and hence assortative mating, if populations with different wintering grounds (i.e., following distinct migratory routes) arrive at different times to their sympatric breeding grounds [14]. However, temporal isolation is rarely strong in populations of migratory birds breeding in the same seasonal habitat because both populations are presumably selected to breed during the peak of food abundance. If post-mating barriers are strong, incipient speciation can occur even with a low degree of assortative mating [15]. A null model suggests that even a ten day difference in arrival date only leads to a moderate degree of assortative mating (30%) among two differently sized blackcap (*Sylvia atricapilla*) populations [16]. This led the authors to conclude that allochrony is not a strong isolating barrier. In general, early and late arriving blackcaps are those that are most likely to mate assortatively [16]. If early arriving blackcaps are in better physical condition (higher body condition and not parasitized) and/or possess higher genetic quality (e.g., are more heterozygous), temporal isolation could be stronger than the null model suggests because early arriving individuals would have higher fitness than later arriving birds. Because temporal variation in proxies of fitness and condition are unknown, the role of temporal isolation in blackcaps remains unresolved.

Distinct migratory routes can not only lead to temporal isolation, but can also be associated with other selection pressures related to different ecological conditions during migration [17]. Ecological conditions such as the abundance of food and parasites can influence arrival times, and hence the degree of temporal isolation, because birds in better physical condition are able to migrate more quickly [18]. For example, blood parasite infection can delay the onset of spring migration and negatively affect body condition in wood-warblers (*Dendroica* spp.) [19]. Currently, it is unknown whether birds with distinct migratory routes differ in physical condition upon arrival on sympatric breeding grounds [14, 16].

The recent establishment of a new migratory polymorphism in central European blackcaps presents a good opportunity to investigate possible mechanisms leading to ecological isolation. Traditionally, blackcaps breeding in southwestern Germany overwinter in the Iberian Peninsula and northern Africa. Since the 1960s, a growing number of blackcaps migrate along a northwesterly route and overwinter on the British Isles [20]. This new migratory direction is genetically determined [21] and highly heritable [22]. Assortative mating occurs between birds with the same migratory direction [14, 16], leading to genetic divergence in sympatric populations [17]. The proximate factors contributing to the rapid microevolution of the new migratory strategy in central European blackcaps are not well resolved. On average, blackcaps overwintering on the British Isles arrive 10 days earlier to the breeding grounds than those overwintering in the Mediterranean region [17]. Given the considerable overlap in arrival timing between birds with different migratory routes [17], it is not possible to unequivocally classify an early arriving individual as being a northwesterly migrant, and respectively, a late arriving blackcap does not necessarily overwinter in the Mediterranean.

Here, we investigate potential factors contributing to premating isolation in blackcaps. To this end, we caught blackcaps in six consecutive years upon their arrival at the breeding grounds in Southwestern Germany and assigned them via stable hydrogen isotope analysis to their wintering grounds. We specifically tested the following hypotheses: (1) Blackcaps with different wintering grounds choose different microhabitats for territory establishment. For this, we analyzed the vegetation structure within blackcap territories using ten different habitat parameters and evaluated via discriminant analysis whether males with different migratory behavior chose different microhabitats for the establishment of their territories. We expect differences in breeding habitat selection as a consequence of either carry-over effects from the wintering area, outcompeting of late arriving individuals from the preferred microhabitat, or imprinting effects. (2) Early arriving migrants have a better physical and genetic condition and higher survival rate than late arriving birds. We used four different proxies for an individual’s fitness and condition: return rates which reliably indicate survival rates in blackcaps [23], body condition, parasite infection and standardized multilocus heterozygosity as proxy for genetic quality. We expected early arriving birds to have better body condition, higher heterozygosity and to be less infected than their later arriving conspecifics, resulting in higher re-sighting and re-capturing rates of these birds in subsequent years.

## Material and Methods

### Study species

The blackcap (*Sylvia atricapilla*) is a migratory warbler common in deciduous forests in the western Palearctic. Sexual dimorphism in the head plumage is pronounced. While males possess a characteristic black cap, females’ head plumage is of a brown color. Upon spring arrival to the breeding grounds (March/April), males choose their territory and start with nest building. Blackcaps are monogamous during the breeding season and with one clutch per year [24]. The female lays between two and seven eggs, that are incubated for about two weeks by both parents [24]. After another two weeks, the chicks are leaving the nest. Between July and October, autumn migration starts [23]. In the breeding population of central Europe, three migratory directions are found: Southwest, southeast and northwest. Whereas the majority of blackcaps migrate either on the southwesterly or southeasterly route to the Mediterranean, an increasing proportion of birds, mainly from southwestern Germany, winters on the British Isles instead [21, 22, 23]. It is supposed that this new strategy is promoted by an increased fitness of birds wintering in Great Britain and Ireland [21].

### Field procedures

We caught blackcaps in six consecutive years (2007–2012) upon their spring arrival on their breeding grounds near Freiburg, (48°00’N, 07°51’E), Southwestern Germany. The blackcap population in this area consists of southwestern migrants wintering in southern France and the Iberian Peninsula as well as northwestern migrants wintering in Great Britain (hereafter SW and NW, respectively). We started catching the first arriving individuals from mid-March each year in our ~50 ha study area in a deciduous forest using standard mist-nets and tape recordings of male blackcap songs as a decoy [16]. Individuals were sexed via plumage differences and weighed (digital balance with 0.1 g precision), then tarsus length was measured (caliper with 0.1 mm precision), which was standardized between multiple observers. Approximately 100 μl of blood were taken from the brachial vein of each individual for genetic analyses and stored at -20°C. We prepared two thin blood smears for each bird (only for the years 2010–2012) for parasite analysis; these were air dried, fixed in 100% methanol and stained with Giemsa in the laboratory. We ringed birds with a standard aluminum ring and a unique combination of three colored rings that enabled us to later identify individuals in the field. We also took samples of the distal claw sections (≤ 2 mm) of most birds to analyze the stable isotope ratio ^2^H/^1^H (denoted as δ^2^H) in the claw tissue. The protocol for handling birds and collecting blood samples at the field site for this study was approved by the Ethics-Commission (University Hospital Freiburg, Hugstetter Str. 55, 79106 Freiburg) and the Regierungspräsidium Freiburg (Referat 35, Veterinärwesen und Lebensmittelüberwachung, Bertoldstr. 43, 79098 Freiburg). To minimize stress, individual birds were handled within less than 10 minutes of capture and released unharmed to their original capture sites. Samples have been collected under field work and animal experiment permits granted by the responsible state environmental offices of Baden-Württemberg (RPT Tierversuch-Nr. 55–8853.17/0).

Each morning, the area was monitored for blackcaps. This enabled us to detect and catch newly arrived individuals. Because we monitored the study area on a daily basis, we considered the day an individual was first captured or seen as a proxy for its arrival date and calculated a day score for each individual where we set the day the first blackcap was caught in a given year as day 1. Spring arrival varied among years (2007: 26 March; 2008: 18 March; 2009: 17 March; 2010: 23 March; 2011: 22 March; 2012: 20 March).

### Stable isotope analysis

All claw tips were cleaned of surface oils in a 2:1 chloroform:methanol solvent rinse and prepared for stable-hydrogen isotope analysis at the Environment Canada stable isotope laboratory in Saskatoon, Canada. Stable-hydrogen isotope analyses of ²H/^1^H (*δ* ^2^H) from claw tips were completed using the comparative equilibration method [25] and measurements were performed on H_2_ derived from high-temperature flash pyrolysis of nails using continuous-flow isotope-ratio mass spectrometry. Measurement of the three keratin laboratory reference materials (CFS, CHS, BWB) corrected for linear instrumental drift were accurate and precise with typical mean *δ* ^2^H ± SD values of –147.4 ± 0.79‰ (*n* = 5), -187 ± 0.56 ‰ (*n* = 5) and -108 ± 0.33 ‰ (*n* = 5), respectively. All *δ* ^2^H measurements were normalized on the Vienna Standard Mean Ocean Water—Standard Light Antarctic Precipitation (VSMOW-SLAP) standard scale. Repeated analyses of hydrogen isotope inter-comparison material IAEA-CH-7 (-100 ‰) and keratin references yielded an (within run) external repeatability of ± 2.0‰.

Across Europe, *δ* ^2^H follows a latitudinal gradient with signatures being more depleted in the north and more enriched in the south. Geographical differences of *δ* ^2^H incorporated in tissues are used to infer wintering origins of migratory birds including blackcaps [14, 16, 26]. We identified blackcaps collected in Freiburg, Germany as SW or NW migrants according to the *δ* ^2^H values of their claw tips. We calculated the ratio of probabilities *p*(r) for each individual to belong to either of the reference distributions *f*(x) or *g*(x).

$$p\left(\text{r}\right)=\frac{f\left(x\right)}{g\left(x\right)+f\left(x\right)}$$

where *f*(x) and *g*(x) are the modeled probability density functions for British and Mediterranean isotope values respectively, calculated for the *δ* ^2^H values from claw tips. Assuming the pools of isotope values to be normally distributed the probability density of *δ* ^2^H values is given by:

$$f\left(x\right)=\;\frac{1}{\sigma \sqrt{2\pi }}exp\left(-\frac{1}{2}{\left(\frac{x-\mu }{\sigma }\right)}^{2}\right)$$

where *σ* is the standard deviation and *μ* the expected value of the respective reference distribution.

For years 2007 and 2008, individuals were assigned to wintering grounds in a previous study [17] utilizing reference SW and NW claw material from blackcaps collected during winters 2006–2008 in southern Spain (mean -70.2 ± 8.1‰) and from tits (*Parus major* and *Cyanistes caerulescens*) caught during winter 2007/2008 in Britain (mean -99.7 ± 9.3‰), respectively. To compensate for annual variation in *δ* ^2^H values during years 2009–2011, replicate samples were standardized between these years. Claw *δ* ^2^H from blackcaps caught in Sevilla, Spain (Jan. 2011) was used as a SW reference (mean -51.6 ± 5.8‰) and claw *δ* ^2^H values of overwintering *Parus* and *Cyanistes sp*. (mean -94.3 ± 4.9‰) [14] as a NW reference for 2009–2011 assignments. Using these parameters, spring blackcaps caught in 2007–2011 in Freiburg were assigned to either one of the NW or SW wintering areas with a confidence of > 75% (Table 1). Birds captured later in the field season (summer and fall) could not reliably be assigned to their wintering quarters because the claw isotopes likely incorporated the local stable hydrogen isotopes of the breeding grounds. We excluded all birds caught later than day 29 in a given year from statistical analysis as day 29 is a highly conservative value to represent isotope values on wintering grounds with 1–2 mm clipped from a claw tip reflecting diet and habitat 2–5 months prior to sampling [27]. Extending the sampling period out another month or more would likely begin to reflect isotope values en route or at breeding grounds.

**Table 1:** Assignments of spring blackcaps caught in Freiburg, Germany from 2007–2011 to NW- or SW-wintering grounds using stable hydrogen isotope (*δ* ^2^H) values from claw tips.

	*n*	mean confidence (± SE)
2007 NW-migrants	9	87.29% (± 4.46)
2007 SW-migrants	73	95.04% (± 0.95)
2007 no assignment	39	
2008 NW-migrants	19	89.63% (± 2.32)
2008 SW-migrants	59	89.62% (± 1.35)
2008 no assignment	17	
2009 NW-migrants	12	99.03% (± 0.62)
2009 SW-migrants	44	99.95% (± 0.05)
2009 no assignment	40	
2010 NW-migrants	16	97.89% (± 1.21)
2010 SW-migrants	121	99.05% (± 0.29)
2010 no assignment	11	
2011 NW-migrants	24	95.97% (± 1.57)
2011 SW-migrants	201	99.56% (± 0.14)
2011 no assignment	25	
2012 no assignment	49	

Samples sizes (*n*) and mean confidences (± standard error) shown for each year. Stable isotopes were not analyzed for 2012 samples.

Mean turnover rate in *δ* ^2^H lies between -0.3‰ and -0.4‰ per day toward breeding ground values [28]. It takes between 95 and 148 days until keratin from the nail base reaches the claw tip, with a mean growth rate of 0.04 ± 0.01 mm per day for palearctic passerines [27]. Mean migration speed of blackcaps in spring is reported as 162 km per day [29], whereas, in general, stopover time is minimized during spring migration because birds are heading toward breeding grounds [30]. So any change en route would have negligible effects on isotope assignment. In total, we caught 759 blackcaps, with 78 belonging to the NW-population and 498 to the SW-population (Table 1). For 183 individuals, no geographical assignment was possible.

### Assessment and comparison of the vegetation structure within territories

In order to investigate a possible relationship between migratory behavior and microhabitat choice, we assessed the vegetation structure of blackcap territories during the breeding season (mid-April to May) for the years 2010 and 2011. We defined a territory as the area where a male was sighted for at least ten consecutive days in company of a female while showing aggressive territorial behavior, e.g. defending against neighboring individuals or intensive singing. The extent of a territory was determined by monitoring the male’s characteristic territorial behavior (e.g., counter-singing). In case the borders were unknown, we defined the territory as a circle with a radius of 20 m around the location where the male was most often seen [31]. We were able to characterize the territories of 36 males which could be identified by their unique color ring combinations; nine of them were classified as NW-migrants and 27 as SW-migrants. We assessed ten parameters of vegetation structure of each territory in mid-June [31, 32] (see Table 2 for parameter descriptions). We used the variables describing the vegetation structure of the territories to evaluate via linear discriminant analysis (LDA) whether males with different migratory behavior chose different microhabitats for the establishment of their territories. Because variables differed in their numerical range, e.g., from 0–100% in “ground cover” compared to 0–30 in “number of ivy trees”, we used z-standardization to standardize all values. As the LDA requires a limited number of variables compared to the sample size in each group, we first conducted a principal component analysis (PCA) for the z-standardized variables and used the first four principal components for the LDA. All statistical analyses were carried out with the free software R v.2.14.1 [33].

**Table 2:** Variables used to assess and compare habitat characteristics of male blackcap territories along with the loadings, eigenvalues and variance of the first four principal components (threshold: 0.4; bold font).

Variables		Loadings			
Habitat variable	Description	PC 1	PC 2	PC 3	PC 4
Ground cover (0–1.5 m)	Percentage of vegetation in this layer	**-0.5364**	0.0587	0.0258	-0.0111
Lower shrubbery layer (1.5–3 m)	Percentage of vegetation in this layer	**-0.5657**	-0.0209	0.0004	-0.1282
Upper shrubbery layer (3–5 m)	Percentage of vegetation in this layer	-0.3116	0.1122	-0.0270	**-0.5348**
Crown layer (above 5 m)	Percentage of vegetation in this layer	0.1165	**0.6804**	-0.0137	-0.0781
Number of ivy trees	Number of trees covered in ivy	-0.0881	**0.7005**	0.0221	0.0700
Nettle covering	Percentage of vegetation covered by nettles	-0.1954	0.0799	-0.0108	**0.8041**
Bramble covering	Percentage of vegetation covered by brambles	-0.0243	0.0146	-0.0147	0.0080
Blooming herbs	Percentage of blooming individuals in herbaceous layer	-0.1265	-0.0993	**-0.6689**	-0.0492
Blooming shrubs	Percentage of blooming individuals in shrubbery layer	**-0.4514**	-0.0630	-0.0311	0.1875
Disturbance	Proportion of the territory that is disturbed (e.g. proximity to highways, noise, walking paths)	0.1240	0.0971	**-0.7410**	0.0472
Eigenvalue		2.183	1.724	1.461	1.336
Variance		27.23	21.50	18.22	16.67

### Arrival date of returning birds

To test our hypothesis that early arriving individuals have a higher survival rate than later arriving birds, we assessed whether re-caught or re-sighted birds arrived earlier on the breeding grounds than the population average. In total, 45 individuals returned to the study area in one or several years after being captured for the first time (9 male SW-migrants, 4 male NW-migrants and 6 female SW-migrants; 23 males and 3 females could not be assigned to one of the two orientations). They were either re-caught or visually identified by their color ring combination. To assess whether the arrival date of returning birds differed from the population mean, we ran a resampling model with 10,000 iterations. Without replacement, we created a random sample of 45 individuals out of the pool of all ringed birds. For each replicate, we calculated the mean arrival date and the 95% confidence interval (interval between 2.5 and 97.5 quantiles of the distribution). If the standard error of the observed mean did not overlap with the confidence interval, we considered the observed value to be significantly different from the expectations of the null model. If the observed mean was outside the confidence interval but standard errors overlapped with it, we considered the observed value to show a trend towards one side of the random distribution. In case the observation was falling inside the 95% confidence interval, it was not considered to be different from the null model.

### Body condition

The body condition of blackcaps caught between 2007 and 2011 (n = 566, n_NW_ = 74, n_SW_ = 492; see S1 Table) was quantified using the scaled mass index [34, 35]. In this index, body mass is standardized at a fixed value of tarsus length, therefore accounting for differences in body size owing to sexual dimorphism and normal growth processes. The scaled mass index thus enables a comparison between individuals of a different age or gender [35]. We assessed the influence of arrival date, sex and migratory direction on body condition and their interactions using covarianve (ANCOVA) models, with year as a random factor. Because the ANCOVA expects the dependent variable to be normally distributed, the values for body condition were square-root transformed to obtain normal distribution. Based on the findings that NW-migrants arrive significantly earlier on the breeding grounds than SW-migrants [16], we tested for correlations between body condition and arrival date separately for the two migratory routes (Kendall correlation). Additionally, we compared the body condition of 78 NW- and 498 SW-migrants with Mann-Whitney U tests.

### Parasite infection

DNA was extracted using the DNeasy Blood and Tissue^®^ Kit (QIAGEN, Hilden). We determined the infection status (haemosporidian parasites) of individuals using PCR (see [36] for methodological details) and microscopy analysis of two blood smears per individual [37]. For microscopy, each blood smear (only samples from 2010, 2011 and 2012) was checked for 25 minutes at low magnification (400x). In total, we obtained data for 302 individuals (n_NW_ = 43, n_SW_ = 195, n_no assignment_ = 64; see S1 Table). A possible relationship between infection status and day of arrival, migration route and the interaction between arrival date and migration route was evaluated using logistic regression. To correct for over-dispersion of data, we used a quasi-binomial family distribution.

### Genetic analysis and heterozygosity

Multi-locus genotypes were obtained for a total of 317 individuals (n_NW_ = 65, n_SW_ = 220, n_no assignment_ = 32; see S1 Table), including 39 re-caught or re-sighted blackcaps (7 male SW-migrants, 4 male NW-migrants and 5 female SW-migrants; 20 males and 3 females could not be assigned to one of the two directions), at 14 microsatellite loci (Syl1, Syl2, Syl6, Syl9, Syl10, Syla1, Syla2, Syla3, Syla12, Syla14, Syla15, Syla18, Syla19, and Syla20) [38, 39]. Standardized heterozygosity [40] (hereafter called “heterozygosity”) was then calculated for each individual. We assessed the influence of arrival date, sex and migratory direction on heterozygosity and their interactions using a covariance (ANCOVA) model with year as a random factor. The relationship between heterozygosity and day score was explored with a Kendall correlation. To test if the mean heterozygosity of re-captured and re-sighted blackcaps was significantly different from that of a random sample of blackcaps, we implemented another null model using the same method as described above: with 10,000 iterations, we drew a random sample without replacement (*n* = 39) and calculated mean heterozygosity as well as the 95% confidence interval and compared the observed value to the model distribution.

## Results

### Vegetation structure within the territories

The first four PCs of the PCA with orthogonal Varimax-rotation accounted for 83.62% of the total variance of the ten variables describing the vegetation structure within territories (Table 2). Original variables with loadings above a threshold of 0.4 were assigned to the respective PC. PC1 mainly characterized density of the vegetation in the lower shrubbery and ground layers as well as the density of blooming shrubs. The number of trees covered in ivy (*Hedera helix*) and the density of the crown layer represented PC2, whereas disturbance and blooming herbs described PC3. PC4 accounted for nettle (*Urtica dioica*) density and the vegetation in the upper shrubbery layer.

**Fig 1:**
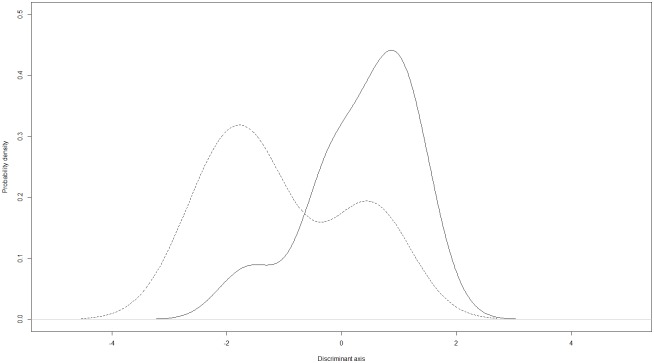
Density functions of the linear discriminant analysis for 2010 and 2011 combined. The functions show the separation of territories of NW-migrants (dashed line) and SW-migrants (solid line) along the discriminant axis. NW-territories occupying the more negative part of the axis were characterized by low density of vegetation in the ground and lower shrubbery layers and low density of blooming shrubs and herbs as well as no disturbance. SW-territories located on the more positive part of the axis showed a high density of nettles, but a low vegetation density in the upper shrubbery layer.

A linear discriminant analysis showed that territories of NW- (n = 9) and SW-migrating males (n = 27) differed in their vegetation structure (Wilks’ Lambda = 0.72, *P* = 0.035; Fig 1). Negative discriminant coefficients characterized territories of NW-migrants, positive coefficients those of SW-migrants. Variables with coefficient values ≤ -0.4 and ≥ 0.4 were considered to contribute strongly to the separation of territories. Territories of NW-migrants were characterized by high values for PC3 and PC1 indicating low density of vegetation in the ground and lower shrubbery layers and low density of blooming shrubs and herbs as well as no disturbance (Table 3). Territories established by SW-migrating males were characterized by high values for PC4, i.e., a high density of nettles, but a low density of vegetation in the upper shrubbery layer (Table 3).

**Table 3:** Linear discriminant coefficients of four PCs for 2010 and 2011 combined and for 2011 separately.

Variables	Linear discriminant coefficients	
	2010 and 2011	2011
PC1	**-0.4026**	**-0.5320**
PC2	0.1504	**1.5295**
PC3	**-0.5794**	-0.3679
PC4	**0.4783**	**1.0326**

Negative values characterize territories of NW-migrants, positive values those of SW-migrants. Variables with coefficient values ≤ -0.4 and ≥ 0.4 (bold font) were considered to contribute strongly to the separation of territories.

To account for year effects, we analyzed the vegetation structure of the territories in a separate LDA for each of the two years. While territories assessed in 2010 (n = 21) did not differ according to the migratory behavior of the occupying male (Wilks’ Lambda = 0.92, *P* = 0.7935), those assessed in 2011 (n = 15) were separated along the discriminant axis (Wilks’ Lambda = 0.30, *P* = 0.0491). Similar to the previous result of both years combined, territories of NW-migrants assessed in 2011 were characterized by high values for PC1, i.e., low density of vegetation in the ground and lower shrubbery layer, whereas SW-territories showed high values for PC2 and PC4, interpreted as a high density of the crown layer and a high number of nettles as well as trees covered in ivy, but a low density of vegetation in the upper shrubbery layer (Table 3).

### Spring arrival

**Fig 2:**
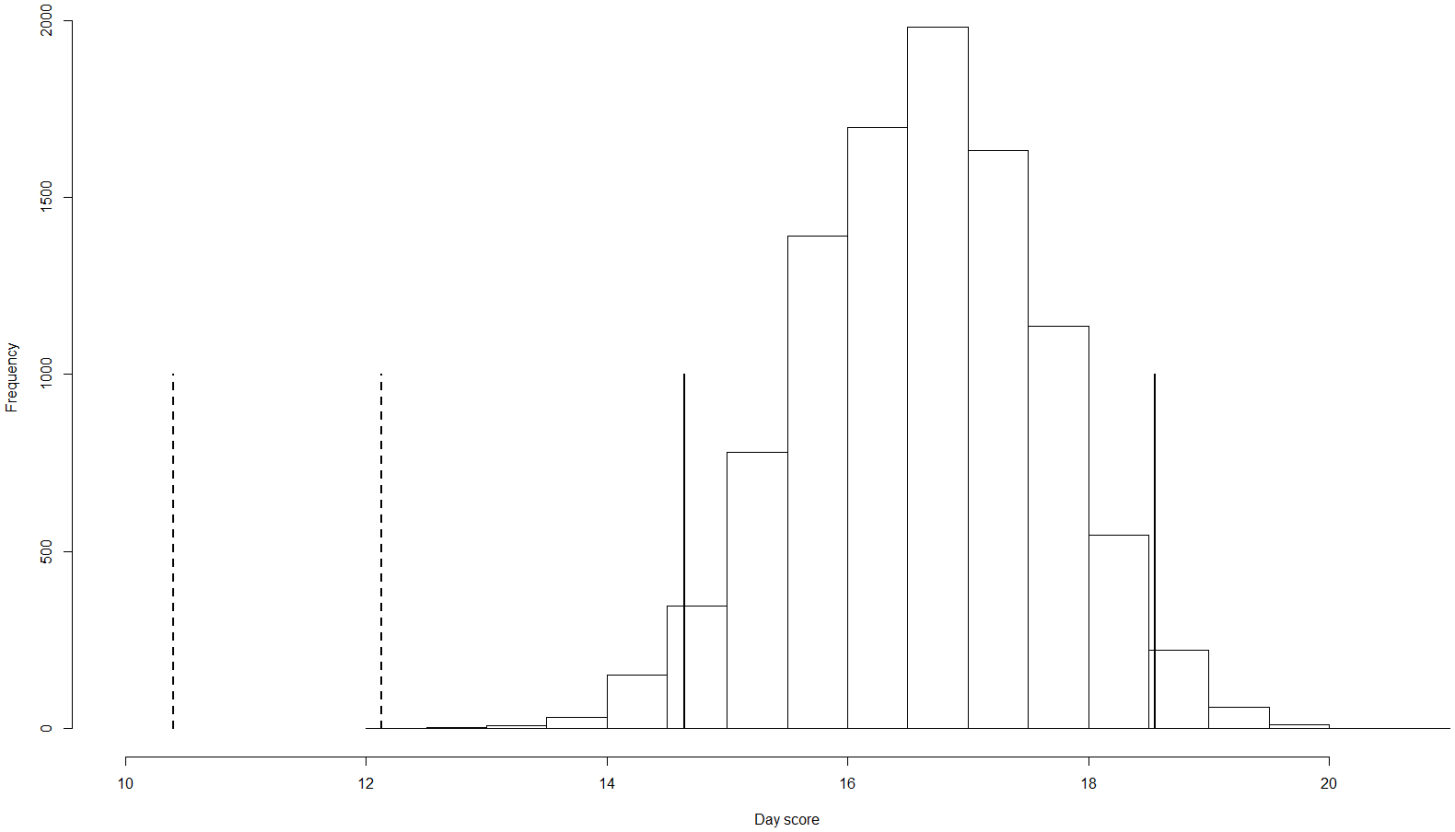
Resampling model of arrival date of re-captured or re-sighted birds and population mean. The histogram presents the frequency of the mean arrival dates of a random sample of 45 individuals out of the pool of all ringed birds, obtained via jackknifing with 10,000 iterations. Standard errors of the observed mean arrival date of returning birds (dashed bars) do not overlap with the 95% confidence interval from the null model (solid bars), indicating that returning birds arrive significantly earlier than the population mean.

Individuals returning to the study site for a second or later year arrived earlier on the breeding grounds than blackcaps undergoing their first migration (Mann-Whitney U test: *n* = 45 vs. *n* = 759, day 11 vs. day 17, *W* = 24,370, *P* < 0.0001). The observed mean arrival date of re-captured or re-sighted individuals (day 11.27 ± 0.86 SE) did not overlap with the confidence interval obtained via jackknifing from all birds that were not re-sighted (mean: day 16.61; 95% CI: 14.62–18.60, Fig 2). We therefore concluded that birds returning in one or several years after being captured for the first time arrived significantly earlier on the breeding grounds than the remaining population.

Body condition influenced the time of arrival (ANCOVA: *P* < 0.0001). The earlier a bird arrived on the breeding ground, the better was its body condition (Kendall correlation: *tau* = -0.220; *P* < 0.0001). Neither migratory orientation (ANCOVA: *P* = 0.216; Mann.Whitney U test: W = 16.529, *P* = 0.202, mean_SW_ = 17.626, mean_NW_ = 17.926), nor sex (ANCOVA: *P* = 0.493), nor the interactions between them (ANCOVA: all *P* > 0.751) affected body condition. As for parasite infection, no significant relationship between an individual’s arrival date and infection status was found (GLM: *t* = -0.729; *df* = 301; *P* = 0.467). Equally, migration route (GLM: *t* = 0.457; *df* = 236; *P* = 0.64) or the interaction between migration route and arrival date (GLM: *t* = -0.118; *df* = 236; *P* = 0.9) did not affect the infection status of blackcaps.

### Heterozygosity

A significant relationship was found between individual heterozygosity and individual day score (ANCOVA: *P* = 0.005; Kendall correlation: *tau* = -0.113, *P* = 0.005), with individuals with earlier dayscores having higher multilocus heterozygosities. Neither migratory direction (ANCOVA: *P* = 0.252; Mann-Whitney U test: W = 7651, *P* = 0.388, mean_SW_ = 0.996, mean_NW_ = 0.972), nor sex (ANCOVA: *P* = 0.131), nor the interactions between them (ANCOVA: all *P* > 0.341) influenced heterozygosity. The observed mean heterozygosity of 39 re-captured and re-sighted blackcaps (1.0716 ± 0.0259 SE) lied outside the confidence interval of the null model distribution (mean: 0.9975; 95% CI: 0.9413–1.0537) with standard errors overlapping with the upper CI. Blackcaps returning in one or several years following initial capture showed a trend towards higher multilocus heterozygosities than the remaining population.

## Discussion

In our study, we analyzed microhabitat preferences, timing of spring arrival and physical as well as genetic condition of blackcaps with different wintering areas upon arrival on sympatric breeding grounds. We found that, depending on their wintering grounds, blackcaps established territories in different microhabitats. Furthermore, early arriving blackcaps were in a better physical and genetic condition than later arriving individuals, leading to the suggestion that temporal isolation can be stronger than previously thought, in particular because it is not just a function of time but likely also of birds’ differential condition over time. Below, we discuss the implications of the observed spatial and temporal segregation for population divergence in blackcaps.

### Habitat segregation

In general, blackcaps prefer to settle in deciduous forests with a high number of trees, whereas they try to avoid open, sunny areas with dense vegetation cover in the shrubbery layer [41]. NW-migrants chose undisturbed areas with low vegetation on the ground and sparsely developed lower shrubbery layer, as well as low density of blooming shrubs and herbs. This vegetation structure is characteristic of undisturbed areas with larger and older trees that build a dense canopy cover suppressing a dense lower shrubbery layer. Territories of SW-migrants, however, had dense nettle cover and low density in the upper shrubbery layer. Nettles, being an indicator plant for nitrogen, are often found in proximity to forest paths or in rather open areas following tree-logging (personal observation). We concluded that SW-migrants establish their territories in disturbed microhabitats that are characterized by sparse vegetation in the upper layer, where less dense canopy and upper shrubbery cover provide the conditions for dense understory vegetation.

The differences in breeding habitat selection of birds with different migratory behavior may result as a consequence of the differences in the timing of spring arrival. The majority of NW-migrants arrive earlier than the SW-migrants [17], so it is conceivable that later arriving SW-migrants are outcompeted from higher quality habitats. The tendency of SW-migrants to settle in more disturbed microhabitats could as well be explained by a carry-over effect from their wintering grounds in southern Spain. Here, two types of habitat are available: forest, which is the preferred wintering habitat, and open scrub, which is less sheltered than forest, but offers a high abundance of food resources [42]. Sedentary Spanish blackcaps occupy forests, where they keep their breeding territories all year round, whereas migrants are found in forests and scrub vegetation [42, 43]. Thus, we hypothesize that birds wintering in scrub vegetation may imprint on this habitat and prefer to breed in more scrubby habitats. Three steps lead to speciation via imprinting on habitat features: first, the colonization of a new habitat; second, genetic divergence of the groups occupying different habitats; and third, a reduction in gene flow between the groups [9]. For blackcaps, the first and second steps apply: we found evidence that NW- and SW-migrants choose different microhabitats, whereas a genetic difference between the two ecotypes has already been reported in previous studies [17, 44]. Whether the observed habitat isolation alone is sufficiently strong to act as a premating barrier to reduce gene flow still remains unclear given the overlap in Fig 1. However, to our knowledge, cross-fostering experiments of blackcaps with different migratory behavior aiming to investigate potential imprinting effects are still absent. Similarly, to firmly establish carry-over effects from habitat choice in winter, tracking of individual birds over the annual cycle would be necessary.

### Temporal isolation

Assortative mating and higher physical condition of blackcaps wintering in Great Britain is considered to be the driving force behind the new migratory strategy [14, 21]. Yet, the previously observed temporal differences in spring arrival of blackcaps did not prove to be sufficiently strong to induce assortative mating and act as a single premating barrier [16]. The model estimates of 30% assortative mating among NW-migrating birds [16] are likely to underestimate assortative mating, as they do not account for differential microhabitat choice (see above) and for fitness differences among blackcaps. Here, we suggest that differing microhabitat preferences act synergistically with temporal isolation due to differences in arrival time and thereby increase the probability of assortative mating of blackcaps with the same migratory route.

Different arrival times in spring are often associated with fitness differences [21]. On average, NW-migrating blackcaps arrive ten days earlier than SW-migrants, with males preceding females in both subpopulations [16]. Our results show that those birds returning in subsequent years were caught on average five days earlier than the mean of the population. The low returning rates of less than 10% for either migratory direction in our study can be attributed to the fact that we only counted re-captured individuals between the years 2008 to 2011. Only in 2012, we additionally counted re-sighted blackcaps. In general, blackcaps are known to show distinctive breeding site fidelity [23], with returning rates of up to 40% [45]. Considering their high mortality rate of about 60% per year, it can be concluded that the majority of the breeding population returns to the previous breeding site [23], making return rates a valuable proxy for the survival rate of blackcaps. Therefore, we suggest that blackcaps arriving early on their breeding grounds indeed have a higher survival rate than birds arriving later in the year. Because the probability of assortative mating among NW migrants is highest early in the season, NW migrants as well as early arriving SW migrants have a stronger effect on population dynamics than null models suggest.

One possible factor contributing to higher survival is physical condition. Our analyses showed that the body condition of early arriving birds is better than that of their later arriving conspecifics. We found no difference in body condition between genders or between migratory routes, which contrasts partly with earlier findings that body condition is influenced only by sex, but not by arrival date or wintering area [16]. This contradiction might be an effect of differences in sample sizes, as we used a much larger dataset, or a sign of annual variation in environmental conditions in the wintering areas. Birds from both wintering grounds have a similar body condition, which indicates that not only food availability in both areas might be similar, but also the flight costs for migration. The fact that both sexes arrive in a similar body condition can as well be attributed to the slight differences in timing of arrival [16]. Even though one would expect that females arrive in a better condition than males for the breeding (breeding performance hypothesis) [46], the fact that males precede females by several days can easily outbalance this relationship: earlier in the season, food sources in the breeding grounds are likely scarce, which is why it is advantageous for males to accumulated large fat resources during spring migration [46]. Still, our findings suggest that early arriving birds are likely to gain selective advantages averaged over longer time spans (and not necessarily in every single year) due to superior physical condition.

Contrary to our expectations, there was no effect of parasite infection on arrival date, leading to the conclusion that blackcaps arriving at different times in early spring to the breeding grounds have a similar infection status. A plausible explanation is that infection with some haemosporidian species is known to have no or only a slightly negative effect on the fitness of their bird host (e.g. *Haemoproteus parabelopolskyi* in blackcaps [47] and some *Plasmodium* genetic lineages in great reed warblers (*Acrocephalus arundinaceus*) [48]). As in a previous analysis, there was no effect of migratory route (NW vs. SW) on infection status [36].

Genetic analyses showed that early arriving individuals as well as blackcaps returning to the study area in one or several subsequent years are more heterozygous than the population mean. Given the positive relationship between heterozygosity and fitness-related traits in birds [49–52], e.g. an increase in clutch size, number of recruits and adult survival [53, 54], we conclude that the early arriving and returning blackcaps are in better genetic condition and likely to show enhanced fitness. Evidence of neutral genetic divergence has already been shown [17], but little is known about the potential for selection against hybrids. Cross-breeding experiments revealed NW x SW hybrids to have intermediate migratory orientations, which is presumed to be maladaptive, leading them to the Atlantic Ocean [22]. In the southwestern German breeding population, it seems that much admixture remains between NW and SW migrants, but the population is beginning to diverge [17, 44]. With sustained divergence of arrival timing and/or microhabitat choice, NW and SW migrants may be expected to further diverge in allele frequencies if hybrids are disadvantageous. We did not carry out tests to determine parentage, as blackcaps are socially monogamous and stay with the same partner even in case the first brood fails. Extra-pair fertilizations are reportedly low [23], reducing the chance of fertilizations by stored sperm of a previous mate drastically. Although we were unable to identify NW x SW hybrids, standardized heterozygosity of the NW migrants is found to be lower (mean 1.040 ± 0.036) than that of SW migrants (mean 1.066 ± 0.017), but it was not found to be different between migratory strategies (Welch’s *t* test: *P* = 0.524). Nevertheless, lower heterozygosity of the NW migrants may indicate increased inbreeding within this group, resulting from positive assortative mating. Higher heterozygosities in the earliest arriving blackcaps may indicate there is a greater proportion of hybrids during this time period.

### Conclusion

We observed different microhabitat preferences on the scale of 50 ha of sympatric blackcaps with distinct migratory routes. Thus, spatial segregation in blackcaps is acting together with temporal isolation. Further, because early arriving blackcaps were more heterozygous, had higher return rates and better physical condition, we suggest that they have a disproportionally high impact on population dynamics. This provides an explanation for why the early arriving NW migrating population has grown since 1960. It further indicates that models on population dynamics in hybrid zones should account for temporal variation in fitness. This is particularly important in migratory divides where migratory routes determine arrival times. Hence, spatial and temporal segregation may act in concert producing assortative mating and unequal fitness consequences which both facilitate ongoing divergence.

## Supporting Information

S1 Table Number of birds used for the different analyses, with their respective sexes and wintering grounds. (DOCX)
