# Supplementary material for: Spatial Isolation and Temporal Variation in Fitness and Condition Facilitate Divergence in a Migratory Divide
Source: PLoS One. 2015 Dec 14;10(12):e0144264. doi: 10.1371/journal.pone.0144264 (PMC4681481; doi:10.1371/journal.pone.0144264)
Supplement: S1 Table — (DOCX) [file pone.0144264.s001.docx]

**Supporting Information**

**S1 Table. Number of birds used for the different analyses, with their respective sexes and wintering grounds.**

|  | Body condition | Parasite infection | Genetic analysis |
| --- | --- | --- | --- |
| 2007 males; NW | 6 | 6 | 6 |
| 2007 females; NW | 3 | 3 | 2 |
| 2007 males; SW | 37 | 37 | 4 |
| 2007 females; SW | 36 | 36 | 6 |
| 2007 males; no assignment |  | 23 | 1 |
| 2007 females; no assignment |  | 15 |  |
| 2008 males; NW | 11 | 11 | 10 |
| 2008 females; NW | 6 | 6 | 4 |
| 2008 males; SW | 38 |  | 2 |
| 2008 females; SW | 21 |  | 1 |
| 2008 males; no assignment |  | 1 | 1 |
| 2008 females; no assignment |  | 1 | 1 |
| 2009 males; NW | 8 |  | 5 |
| 2009 females; NW | 4 |  | 1 |
| 2009 males; SW | 12 |  | 8 |
| 2009 females; SW | 32 |  | 22 |
| 2009 males; no assignment |  |  | 2 |
| 2009 females; no assignment |  |  | 5 |
| 2010 males; NW | 10 | 10 | 9 |
| 2010 females; NW | 6 | 6 | 6 |
| 2010 males; SW | 64 | 64 | 49 |
| 2010 females; SW | 57 | 57 | 44 |
| 2010 males; no assignment |  | 6 | 5 |
| 2010 females; no assignment |  | 4 | 2 |
| 2011 males; NW | 12 |  | 13 |
| 2011 females; NW | 8 | 1 | 9 |
| 2011 males; SW | 71 |  | 29 |
| 2011 females; SW | 124 | 1 | 55 |
| 2011 males; no assignment |  | 10 | 10 |
| 2011 females; no assignment |  | 1 | 2 |
| 2012 males; no assignment |  | 3 | 3 |
| 2012 females; no assignment |  |  |  |
| Sum | 566 | 302 | 317 |
